# A Plasma Biochemical Analysis of Acute Lead Poisoning in a Rat Model by Chemometrics-Based Fourier Transform Infrared Spectroscopy: An Exploratory Study

**DOI:** 10.3389/fchem.2018.00261

**Published:** 2018-06-28

**Authors:** Wenli Tian, Dan Wang, Haoran Fan, Lujuan Yang, Gang Ma

**Affiliations:** Key Laboratory of Medicinal Chemistry and Molecular Diagnosis of Ministry of Education, Key Laboratory of Analytical Science and Technology of Hebei Province, College of Chemistry and Environmental Science, Hebei University, Baoding, China

**Keywords:** FTIR spectroscopy, infrared spectroscopy, chemometrics, lead poisoning, acute lead poisoning, principle component analysis, partial least squares discriminant analysis

## Abstract

In this work, we explored to use chemometrics-based Fourier transform infrared (FTIR) spectroscopy to investigate the plasma biochemical changes due to acute lead poisoning (ALP) in a rat model. We first collected the FTIR spectra of the plasma samples from the rats with and without suffering from ALP. We then performed the chemometric analysis of these FTIR spectra using principal component analysis (PCA) and partial least squares discriminant analysis (PLS-DA). We found that the chemometrics-based FTIR spectroscopy can discriminate the rats with and without ALP. Further analysis on the PLS-DA regression coefficient revealed that the spectral changes, in particular, corresponding to the biochemical changes of proteins in the plasma may be used as potential spectral biomarkers for the diagnostics of lead poisoning. Our work demonstrates the potential of chemometrics-based FTIR spectroscopy as a promising tool for the biochemical analysis of plasma that could consequently enable an objective, convenient and non-destructive diagnostics of lead poisoning. To the best of our knowledge, this work is the first application of chemometrics-based FTIR spectroscopy in the diagnostics of lead poisoning.

## Introduction

Lead is an omnipresent metal that has been used since prehistoric times. Prior to the industrial revolution, human exposure to lead in the environment was relatively low, but significantly increased over time due to modern industrial activities. It is estimated that over 300 million tons of lead has been released to the environment by human activities (Tong et al., 2000), which leads to a rapid increase in lead exposure to the environment. A previous study indicated that the lowest levels of human blood lead in industrial era were 50–200 times higher than preindustrial era (Flegal and Smith, 1992b). As for lead poisoning, in 1839, Tanqueral des Planches described the symptoms of acute lead poisoning (ALP) and studied the signs of ALP in adults (Hunter, 1978). In the middle and late nineteenth century, lead poisoning became a serious health problem among Britain workers. British Parliament eventually enacted relevant laws and regulations to prevent lead poisoning (Hunter, 1978; Smith, 1984; Winder, 1984; Tong et al., 2000). Lead poisoning can be caused by human ingestion and respiration of lead and related products such as lead-containing paints. Lead can cause a series of physiological and biochemical changes within human body, affecting central and peripheral nervous system, cardiovascular system, reproductive system, immune system, gastrointestinal tract, liver, kidney and brain (Hunter, 1978; Smith, 1984; Winder, 1984; Kazantzis, 1989; Goldstein, 1992; Tong, 1998; Tong et al., 2000).

The basic principle in lead poisoning diagnostics is based on the determination of lead level in human body. There are currently several methods available for measuring lead in blood samples. For example, one common method is the so-called blood film method, in which the morphology of the red blood is examined with a microscope to reveal basophilic stippling of red blood cells (i.e., red blood cells with dots in their morphologies). However, this method is not very specific because other unrelated conditions (such as folate and vitamin B12 deficiencies) can also give basophilic stippling of red blood cells. Lead level can be evaluated indirectly by measuring erythrocyte protoporphyrin (EP) in blood samples. It is noted that such EP measurement is not very sensitive and specific because an increase in EP level can also be observed in the case of iron deficiency. X-ray fluorescence method can be used to determine the cumulative exposure and total body burden of lead. However, this method is not so convenient because X-ray fluorescence instrument is not widely available in clinic. Apparently, the current methods in lead poisoning diagnostics still have some limitations and disadvantages (Patrick, 2006; Brodkin et al., 2007). Searching for a specific, rapid, convenient, objective and cost-effective method for lead poisoning diagnostics is no doubt very meaningful (Flegal and Smith, 1992a).

In recent years, Fourier transform infrared (FTIR) spectroscopy has been widely used in the biochemical analysis field (Baker et al., 2014). FTIR spectroscopy is a simple, convenient, non-destructive, rapid and low-cost detection method to sample biological materials such as blood and tissue for diagnostic purposes (Deleris and Petibois, 2003; Ellis and Goodacre, 2006; Krafft et al., 2007, 2009; Gasper et al., 2009; Gajjar et al., 2013; Baker et al., 2014; Mitchell et al., 2014; Ollesch et al., 2014; Sheng et al., 2015; Staniszewska-Slezak et al., 2015; Depciuch et al., 2017; Elmi et al., 2017; Ghimire et al., 2017; Guo et al., 2017; Le Corvec et al., 2017; Li et al., 2017; Liu et al., 2017; Paraskevaidi et al., 2017; Roy et al., 2017; Sarkar et al., 2017; Titus et al., 2017; De Bruyne et al., 2018; Rai et al., 2018). When combined with chemometric analysis, FTIR spectroscopy can be further empowered in disease diagnostics. Now, FTIR spectroscopy has been used in many studies to detect the physiological states and disease-specific biomarkers in the blood. For example, Staniszewska-Slezak et al. established the rat models for pulmonary arterial hypertension and systemic hypertension, and then collected the FTIR spectra of rat plasma samples. By using FTIR spectroscopy combined with principal component analysis (PCA), they found that they could distinguish the two different hypertension states as well as the healthy state. They also envisioned that chemometrics-based FTIR spectroscopy could potentially provide some spectral biomarkers for disease diagnostics (Staniszewska-Slezak et al., 2015). Roy et al. recently used attenuated total reflection Fourier transform infrared (ATR-FTIR) spectroscopy in combination with partial least squares discriminant analysis and partial least squares regression to identify malaria parasites, blood glucose and urea levels in whole blood samples (Roy et al., 2017). Titus et al. recently proposed an FTIR approach combined with cluster and heterogeneity analyses to rapidly screen colitis without using biopsies or *in vivo* measurements (Titus et al., 2017). Paraskevaidi et al. recently demonstrated an excellent diagnostic performance of chemometrics-based ATR-FTIR spectroscopy by analyzing plasma samples from patients with Alzheimer's disease (Paraskevaidi et al., 2017).

In our work, we focused on the biochemical changes of plasma after lead poisoning using a rat model suffering from ALP. The main goal of this study was to find the plasma biochemical changes induced by lead in rats by FTIR spectroscopy combined with chemometric approaches such as PCA and partial least squares discriminant analysis.

## Experimental

### ALP rat model

Male Wister rats (240 ± 20 g) were purchased from the Vital River Lab Animal Technology Co., Ltd. (Beijing, China). Animals were housed under constant temperature, humidity and lighting (12 h per day) and were allowed free access to food and water. The animal experiment was carried out in accordance with the guidelines for the care and use of laboratory animals and the relevant ethical regulations of the Animal Ethics Committee of Tianjin Tasly Institute. The protocol was approved by the Animal Ethics Committee of Tianjin Tasly Institute.

The rats (*N* = 4) before lead injection were used as the control group and these rats after lead injection used as the test group. To induce ALP, the rats were intraperitoneally injected with PbCl_2_ saline solution (5 mg lead per kg). For chemometric modeling, blood samples were collected from the control group and the test group 24 h post-injection. Blood samples were also collected from the test group 36 and 48 h post-injection for model validation. In addition, another control group (*N* = 4), namely a group with acute cadmium poisoning, was studied by intraperitoneally injecting the rats with CdCl_2_ saline solution (5 mg cadmium per kg). The blood samples from this control group were collected 24 h post-injection. The blood samples were stored at about −80 °C for further treatment. Both PbCl_2_ and CdCl_2_ of analytical grade were obtained from local vendors.

### Plasma sample preparation

The blood sample was centrifuged at 3,000 rpm for 10 min, and a 10-μl aliquot of supernatant plasma was pipetted on the top of a piece of 1 × 1 cm aluminum foil. Each blood sample was used to prepare five replicate samples on aluminum foil. The foil was then placed in an oven set at 37°C for 2 h, and the obtained dry plasma film was subsequently used for FTIR measurement.

### FTIR measurement

FTIR measurements were carried out on a Bruker Vertex 70 FTIR spectrometer (Ettlingen, Germany) equipped with a DLaTGS detector in attenuated total reflection (ATR) mode. 4 cm^−1^ resolution and 32 scans were used for each measurement. A Pike Technologies MIRacle single-reflection ATR accessory (Madison, USA) with a diamond element was employed. When performing spectral acquisition, the plasma sample was pressed against the diamond crystal using a pressing device from Pike Technologies for a close contact. For each piece of aluminum foil with blood sample, at least seven FTIR spectra were taken by measuring signals at different locations on the foil.

### Spectral pretreatment

The obtained FTIR spectra of the plasma samples were first screened to remove some error-based large deviation spectra. In ATR-FTIR mode, the contact between the sample and diamond crystal has a significant effect on the spectral quality, e.g., a poor contact will lead to poor quality FTIR spectra (abnormally low absorbance). These spectra need to be removed from the spectral dataset before chemometric analysis. Such spectral deviation is not due to the intrinsic deviation of one sample from its group (i.e., the control or test groups), but purely related to the spectral artifact caused by an improper contact between the sample and diamond crystal. These “abnormal” spectra could be easily identified visually with OPUS software and they were then removed from the spectral dataset manually. The remaining spectra were used for chemometric analysis after being subjected to spectral pre-treatment including smoothing, scattering correction, vector normalization and second derivative treatment with chemometric software.

### Chemometric analysis

Chemometric analysis was performed using Unscrambler software (version 10.4) for PCA and partial least squares discriminant analysis (PLS-DA). In our study, we selected the data from the second derivative FTIR spectra in the regions of 3,100–2,800 and 1,750–900 cm^−1^ for PCA. In addition, we also used 4-fold cross validation to test rat inter-individual variability on the spectra. The above-mentioned chemometric approach is relatively simple and sufficiently powerful to help differentiate the rat groups with and without ALP, spectroscopically.

## Results and discussion

**Figure 1:**
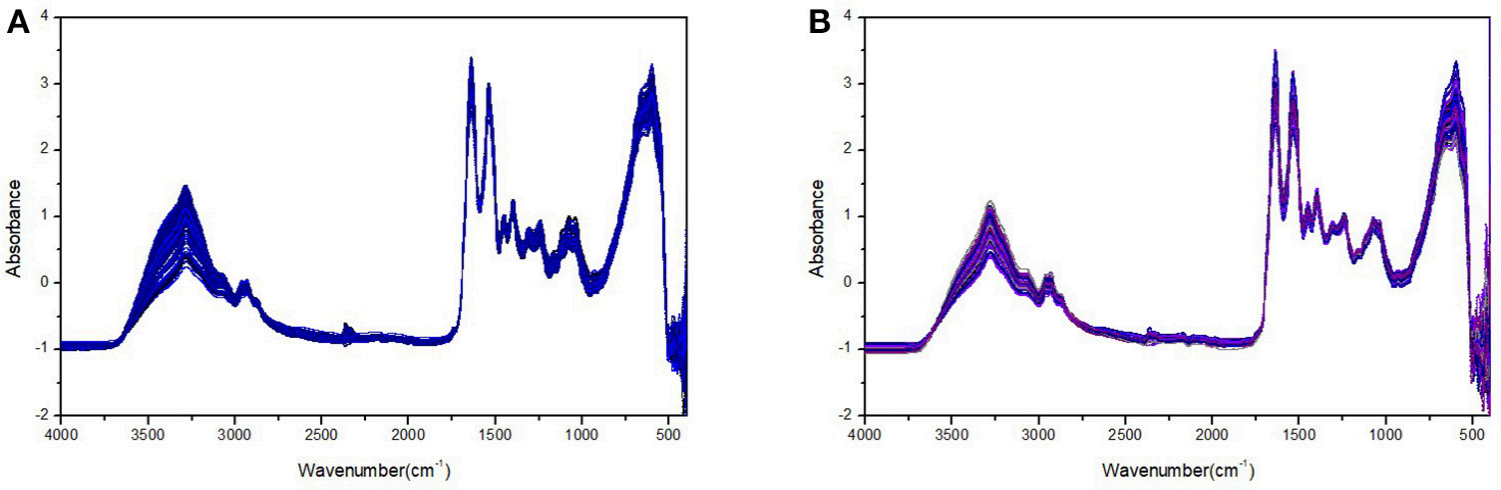
Plasma FTIR spectra of the rat group without ALP **(A)** and with ALP **(B)** after spectral pretreatment such as smoothing, baseline correction, and vector normalization. The spectra with ALP were collected 24 h post-injection and there are a total of 139 spectra included in **(A)** and a total of 125 spectra included in **(B)**.

**Figure 2:**
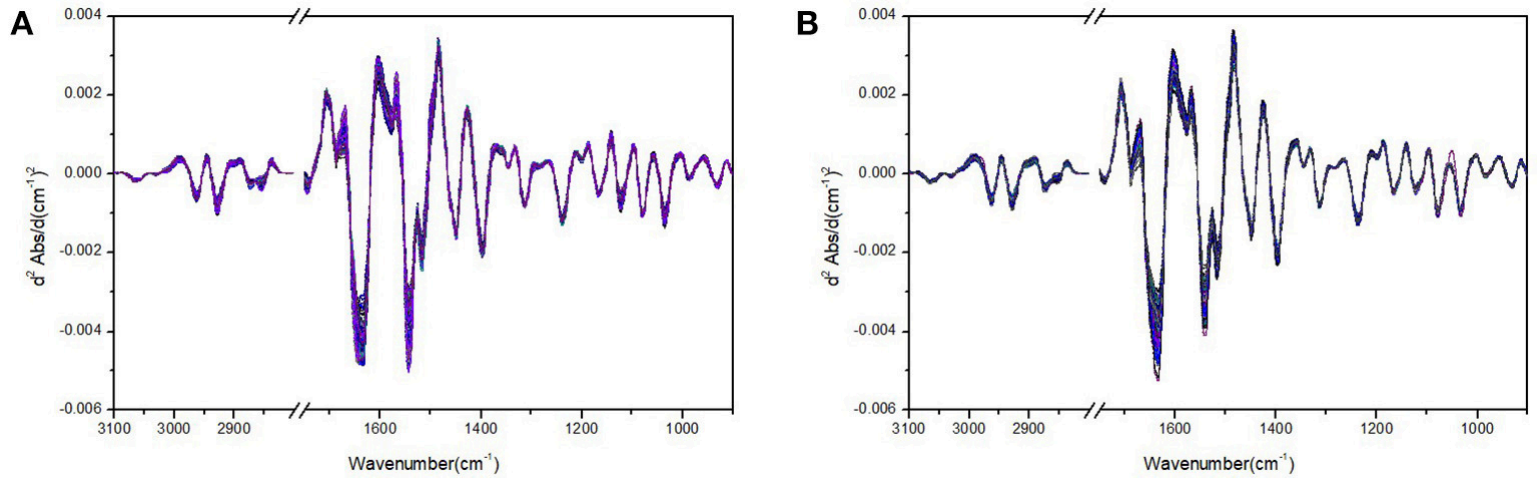
Plasma FTIR second derivative spectra of the rat group without ALP **(A)** and with ALP **(B)** in the 3,100–2,800 and 1,750–900 cm^−1^ spectral regions.

Figure 1 shows the plasma FTIR spectra of the rat groups without and with ALP after spectral pretreatment such as smoothing, baseline correction, and vector normalization. On the other hand, Figure 2 shows the second derivative spectra of the plasma FTIR spectra presented in Figure 1. These second derivative spectra were the dataset used in the following chemometric analysis. The reason to have derivative treatment on the absorbance spectra in Figure 1 is 2-fold. First, the second derivative treatment can further magnify the spectral changes and differences between the control and test groups. Second, the second derivative treatment can also eliminate possible interference of the baseline in chemometric analysis. In addition, in Figure 2, we have only included the spectral regions of 3,100–2,800 and 1,750–900 cm^−1^ and removed the spectral region of 2,800–1,750 cm^−1^ (as this region contains very limited spectral information). The 3,100–2,800 cm^−1^ region corresponds to the C-H stretching absorptions; whereas the 1,750–900 cm^−1^ corresponds to the protein amide I and amide II regions, and the fingerprint region. The displayed spectral regions in Figure 2 contain most of the spectral information that is highly correlated to the ALP-induced biochemical changes in the plasma, thus making them suitable in our chemometric analysis.

As for the control and test group spectra datasets, we first used the most basic chemometric approach, PCA, to perform data analysis. We found the contribution rates of the first five principal components (namely PC-1, PC-2, PC-3, PC-4, and PC-5) are 64, 20, 7, 3, and 2%, respectively. The cumulative contribution rate of these five principal components reaches 96%, indicating that they can reflect most of the spectral variations and differences among the spectra of the control and test groups.

**Figure 3:**
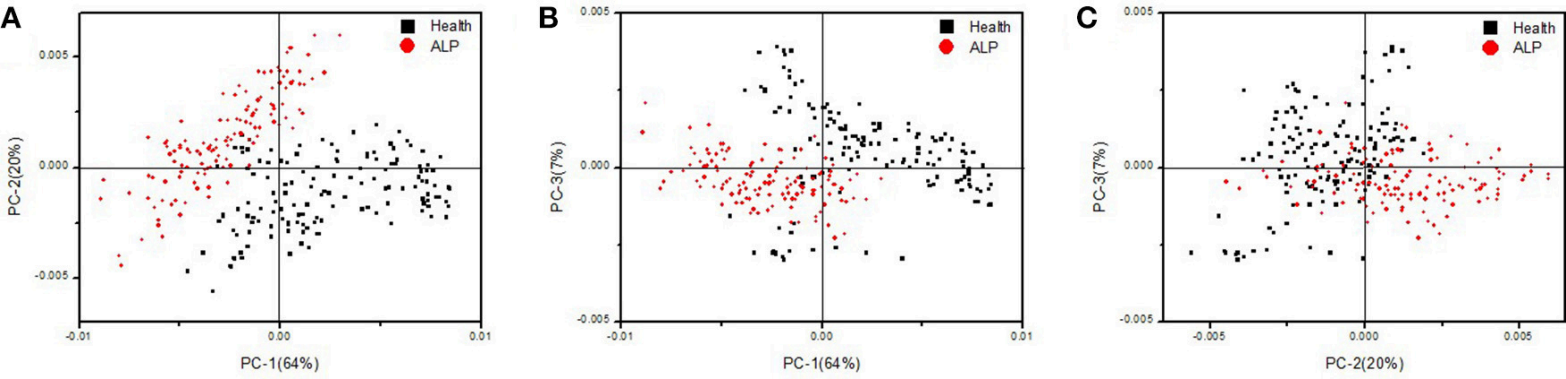
Two-dimensional score plots of PC-1 vs. PC-2 **(A)**, PC-1 vs. PC-3 **(B)**, and PC-2 vs. PC-3 **(C)** obtained after PCA applied to the FTIR second derivative spectra of the rat groups without and with ALP.

The two-dimensional score plots of PC-1 vs. PC-2, PC-1 vs. PC-3 and PC-2 vs. PC-3 were respectively shown in Figures 3A–C. Among the three score plots, we can clearly see that the two groups are well separated (Figure 3A) or they still have some significant overlaps (Figures 3B,**C**). Figure 3A gives the best discriminant result for the control and test groups. Our chemometric analysis study obviously demonstrates that with just some simple chemometric approaches such as PCA and PLS-DA, FTIR spectroscopy can be used to discriminate the rat groups with and without ALP.

**Figure 4:**
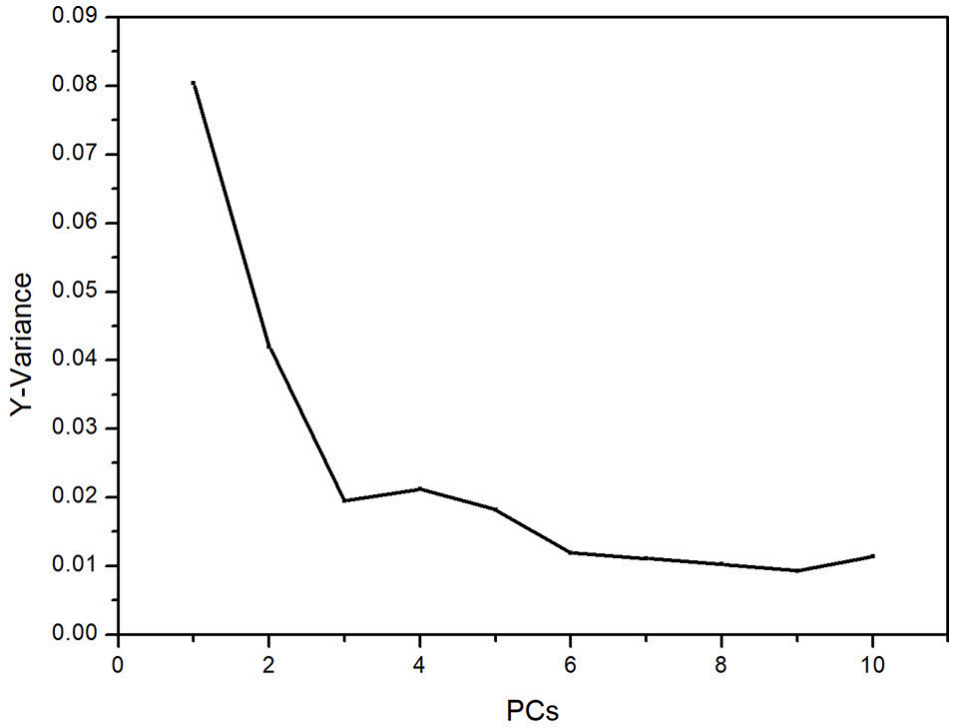
PLS-DA Y-variance plot in the 3,100–2,800 and 1,750–900 cm^−1^ spectral regions.

**Figure 5:**
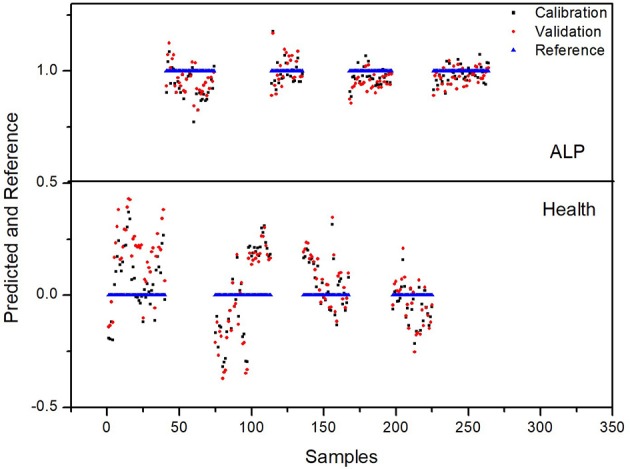
PLS-DA predicted and reference plots in the 3,100–2,800 and 1,750–900 cm^−1^ spectral regions.

**Figure 6:**
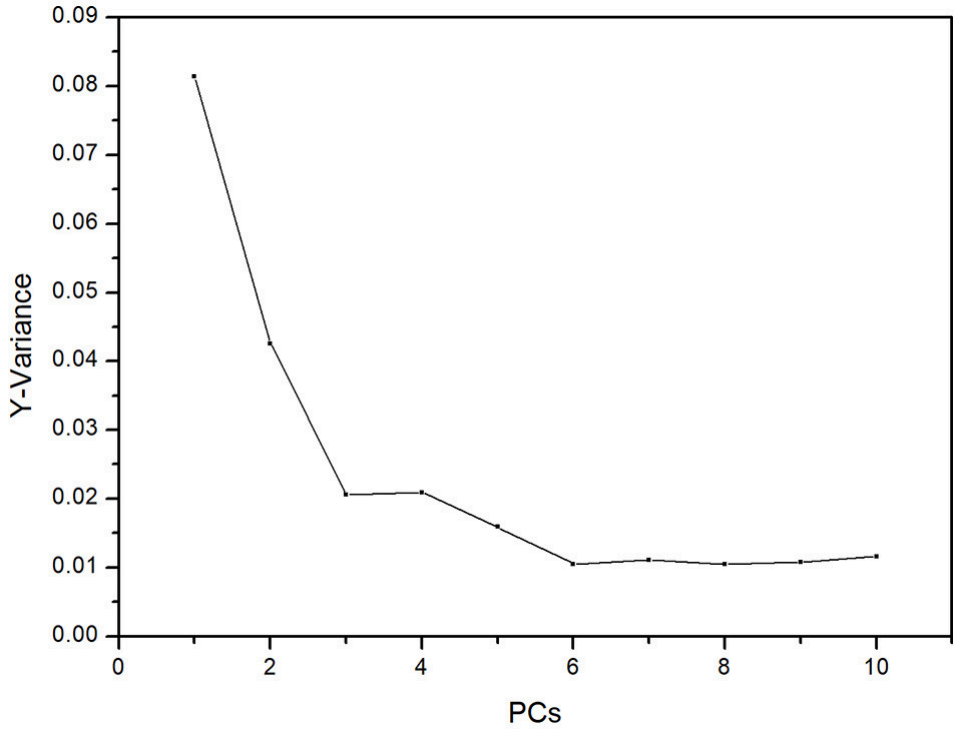
PLS-DA Y-variance plot in the 1,750–900 cm^−1^ spectral region.

**Figure 7:**
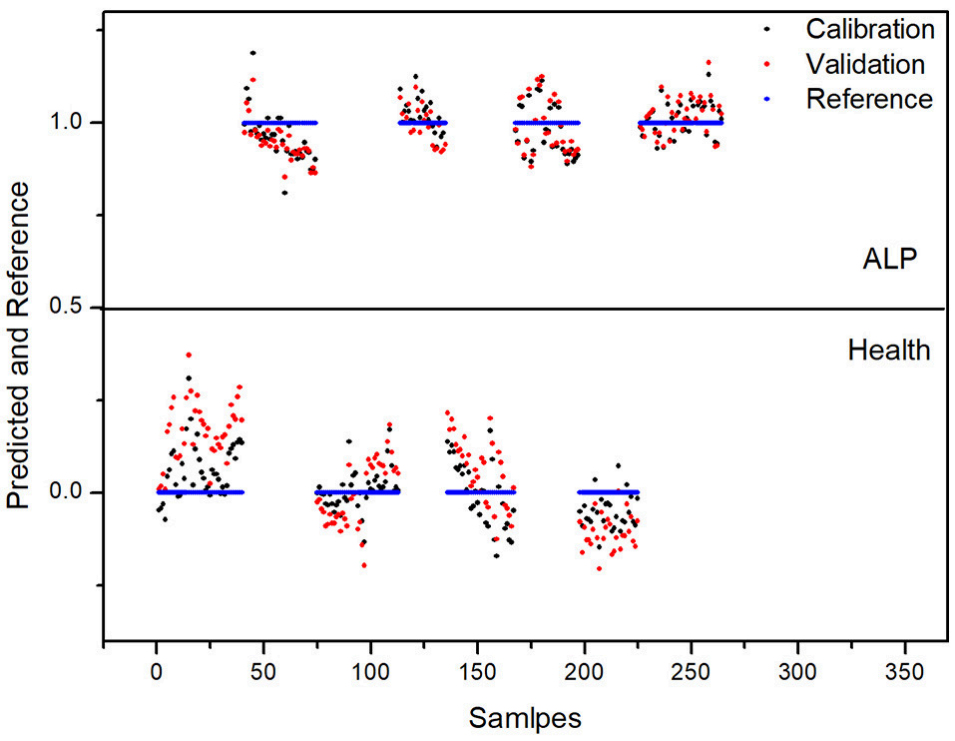
PLS-DA predicted and reference plot in the 1,750–900 cm^−1^ spectral region.

For 4-fold cross validation on our data, each sample was used once as a test set while the remaining samples formed the training set. The results show that (i) there are significant differences between the test and control groups of plasma due to ALP and (ii) rat inter-individual variability has little influence on the spectral differences between the two groups. First, we analyzed the regions of 3,100–2,800 and 1,750–900 cm^−1^ with PLS-DA. As displayed in Figure 4, the Y-variance plot shows that the line was basically leveled at PC7, and the more PCs could be overfitting; so seven PCs were selected for further analysis. Figure 5 shows that PLS-DA could distinguish between health and ALP rats completely with seven PCs. However, the blue and red models of cross validation (CV) were not well matched. So, the fingerprint region of 1,750–900 cm^−1^ was selected. As displayed in Figure 6, the Y-variance plot shows that seven PCs should be selected for further analysis. Figure 7 shows not only that PLS-DA can distinguish between health and ALP rats completely with seven PCs, but also that the blue model fits well with the red CV model. In addition, the health and ALP groups in the red CV model are well separated by the 0.5 threshold line. In summary, the plasma spectra of health and ALP rats were distinctly different and inter-individual variability had no impact on the discrimination analysis of health and ALP rats.

**Figure 8:**
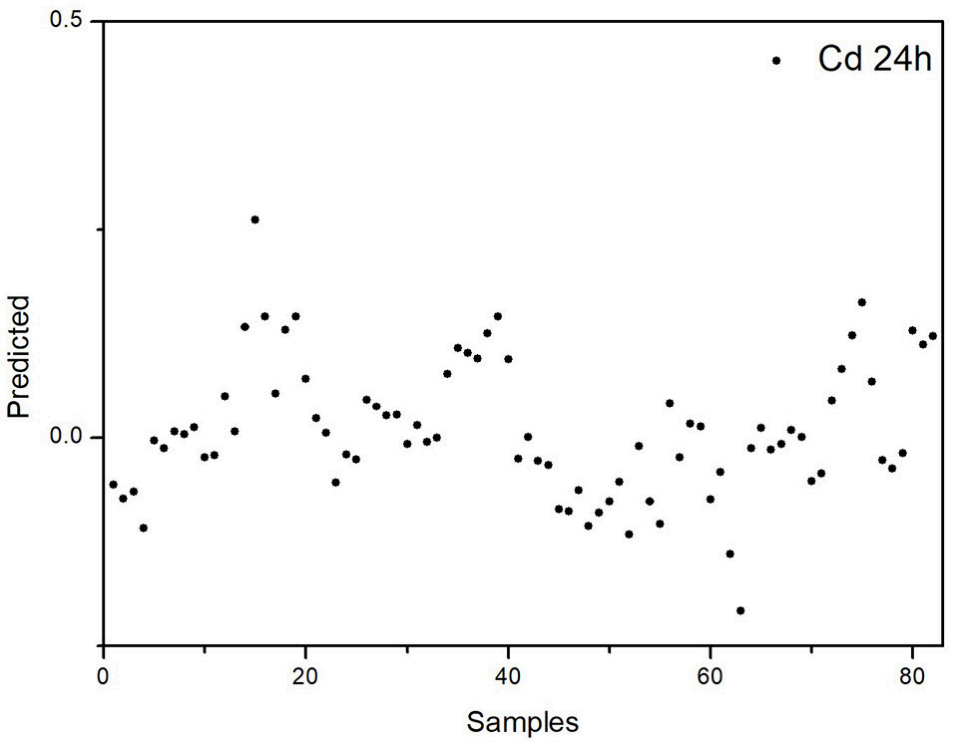
Discrimination of the rats with acute cadmium poisoning using the proposed PLS-DA model for ALP.

**Figure 9:**
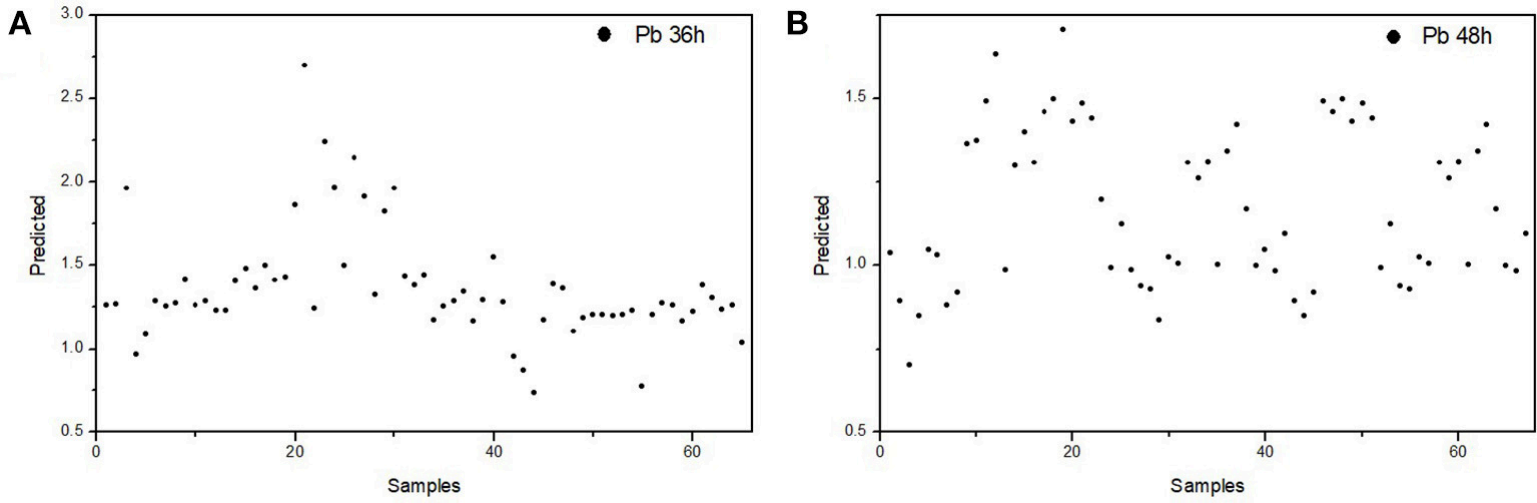
Discrimination of the rats suffering from different extents of ALP using the proposed PLS-DA model: **(A)** 36h post-injection; **(B)** 48h post-injection.

The selectivity and robustness of our proposed PLS-DA model were also tested with additional controls to evaluate whether this model can give a correct discrimination when (i) when the rats suffer from another heavy metal poisoning and (ii) when the rats suffer from different extents of ALP. To address the first issue, we developed an acute cadmium poisoning rat model. Rats were injected with CdCl_2_ solution to induce acute poisoning and the blood samples were collected 24 h post-injection. The plasma FTIR spectra and corresponding derivatives of this control group are presented in Figure S1 in the Supplementary Material. The data with this control group were tested with our PLS-DA model. As we have mentioned above, the 0.5 value line is the threshold in the PLS-DA model in Figure 7. For data points above this line, the model predicts the rats are in ALP status; for data points below this line, the model predicts the rats are not in ALP status. As displayed in Figure 8, the predicted values for the rats suffering from acute cadmium poisoning are all below the 0.5 threshold, indicating that our PLS-DA model predicts that the rats suffering from cadmium poisoning are not in ALP status. This is a correct discrimination. To address the second issue, we performed a time-dependent study (up to 48 h post-injection) on the ALP rat model. The rats exposed to lead poisoning for different periods of time would suffer from lead poisoning to different extents. The plasma FTIR spectra and corresponding derivatives of this control group are presented in Figures S2, S3 in the Supplementary Material. We tested the 36 and 48 h data with our PLS-DA model. As we can see in Figure 9, the predicted values for these two control rat groups are all above the 0.5 threshold, indicating that these samples are in ALP status. This is a correct discrimination. These additional control experiments support the fact that our PLS-DA model is robust for ALP prediction.

**Figure 10:**
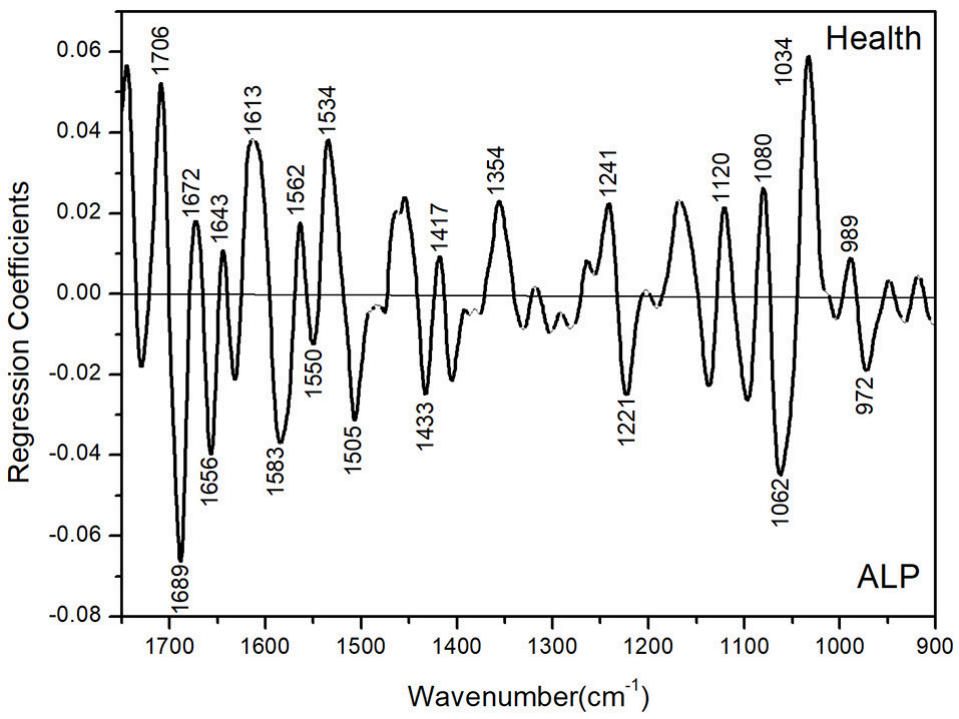
PLS-DA regression coefficient plot in the 1,750–900 cm^−1^ spectral region.

Basically, some lead-induced biochemical changes in the plasma can be sensitively captured with chemometrics-based FTIR spectroscopy. To gain more insight into the biochemical changes induced by ALP in the plasma, the PLS-DA regression coefficient plot could be used to reflect corresponding spectral changes. As shown in Figure 10, this plot corresponds to the ALP-induced change in the composition and structure of the biochemical components in the plasma including biomacromolecular constitutes (such as proteins, DNAs and RNAs) as well as small molecular constitutes and metabolites (such as lipids and carbohydrates). These plasma constitutes have characteristic vibrational absorptions in the PLS-DA regression coefficient plot. For example, through the spectral analysis of the 1,700–1,600 cm^−1^ amide I region, we could obtain the information relevant to proteins; through the spectral analysis of the 1,300–1,000 cm^−1^ region, we could obtain the information relevant to DNA and RNA. In addition, the intensity of the PLS-DA regression coefficient plot in different spectral regions could also provide information about the most prominent changes in the plasma. A summary is provided in Table 1 for the spectral assignments for prominent peaks (either positive or negative) in the PLS-DA regression coefficient plot. They are based on the assignments in previous studies (Barth and Zscherp, 2002; Zandomeneghi et al., 2004; Zou et al., 2013; Staniszewska-Slezak et al., 2015). The peaks in the amide I (1,700–1,600 cm^−1^) and amide II (around 1,550 cm^−1^) correspond to absorptions of plasma proteins. In this region, we observed several prominent peaks in the PLS-DA regression coefficient plot including the amide I and amide II peaks at 1,706, 1,689, 1,672, 1,656, 1,643, 1,613, 1,550, and 1,534 cm^−1^. This observation in the PLS-DA regression coefficient plot suggests that ALP induced significant compositional and structural changes of the proteins in the plasma of the ALP rat model. Such changes may be due to the direct coordination effect of lead ion with protein or be due to the perturbation of lead ion on the biosynthesis of proteins in the rat. In addition, lead ion may interact (or coordinate) with the side chains of some amino acids (such as tryptophan, histidine, aspartic acid, and glutamic acid) or affect the biosynthesis of these amino acids. Such interactions or perturbations are suggested by the observation of the peaks at 1,505, 1,354, and 1,241 cm^−1^ (corresponding to the side chain of tryptophan), at 1,583 and 1,433 cm^−1^ (corresponding to the side chain of histidine) and at 1,417 cm^−1^ (corresponding to the side chains of aspartic acid and glutamic acid). The PLS-DA regression coefficient plot also suggests that the nucleic acid, DNA and RNA changes in the plasma as the peaks at 1,221, 1,120, 1,080, and 1,062 cm^−1^ are observed in the regression coefficient. These peaks correspond to the ${\text{PO}}_{2}^{-}$ and C-O absorption of DNA and RNA. At last, the peaks at 1,034 cm^−1^ (which may be related to the metabolism of glucose and polysaccharides) and at 989 and 972 cm^−1^ (which corresponds to the phosphorylation modification of proteins) are also observed in the regression coefficient plot. In summary, on the one hand, the PLS-DA regression coefficient plot suggests a very complex biochemical changes that occurred in the body of the lead-poisoned rats; one the other hand, ALP-induced protein changes seem to be the most important cause for the rat poisoning. This finding further implies that the spectral changes corresponding to the biochemical changes of proteins may be used as potential spectral biomarkers for the diagnostics of ALP.

**Table 1:** Spectral assignment for the observed peaks in the PLS-DA regression coefficient plot.

**Peak position (cm^−1^)**	**Spectral assignment**
1,706	Protein amide I
1,689	Protein amide I
1,672	Protein amide I
1,656	Protein amide I
1,643	Protein amide I
1,613	Protein amide I
1,583	C = C vibration of histidine
1,562	Protein amide II
1,550	Protein amide II
1,534	Protein amide II
1,505	Indole vibration of tryptophan
1,433	C-N vibration of histidine
1,417	C-C, C-H, and N-H vibrations of tryptophan
1354	Indole vibration of tryptophan
1,241	C-H and C-C vibrations of tryptophan
1,221	${\text{PO}}_{2}^{-}$ antisymmetric stretch of nucleic acids, DNA, and RNA
1,120	C-O stretch of DNA and RNA
1,080	${\text{PO}}_{2}^{-}$ vibrations of nucleic acids, phospholipids, and saccharids
1,062	${\text{PO}}_{2}^{-}$ symmetric stretch of nucleic acids, DNA, and RNA
1,034	C-O-H bend of glucose and polysaccharide
989	Protein phosphorylation
972	Protein phosphorylation

## Conclusion

In this exploratory study, we have demonstrated that FTIR spectroscopy empowered with PCA and PLS-DA analysis can capture ALP-induced biochemical changes in the plasma spectroscopically and is capable of differentiating the rats with and without suffering from ALP. Furthermore, the revealed FTIR spectral changes, in particular, corresponding to the biochemical changes of proteins, may be used as potential spectral biomarkers for the diagnostics of lead poisoning. Our method has sufficient discriminant ability and the potential to be employed as a blood-based objective, convenient, and non-destructive diagnostic tool for lead poisoning. To the best of our knowledge, this work is the first application of chemometrics-based FTIR spectroscopy in the diagnostics of lead poisoning. We hope the chemometrics-based FTIR spectroscopy can evolve into an objective, convenient, cost-effective and non-destructive disease diagnostics tool in the future.

## Ethics statement

This study was carried out in accordance with the recommendations of institutional guidelines of the Animal Ethics Committee of Tianjin Tasly Institute. The protocol was approved by the Animal Ethics Committee of Tianjin Tasly Institute.

## Author contributions

WT and GM designed the project. WT, DW, HF, LY, and GM conducted the experiments and analysed the data. GM, WT, and HF wrote the manuscript.

### Conflict of interest statement

The authors declare that the research was conducted in the absence of any commercial or financial relationships that could be construed as a potential conflict of interest.
