# Supplementary material for: A Plasma Biochemical Analysis of Acute Lead Poisoning in a Rat Model by Chemometrics-Based Fourier Transform Infrared Spectroscopy: An Exploratory Study
Source: Front Chem. 2018 Jun 28;6:261. doi: 10.3389/fchem.2018.00261 (PMC6031737; doi:10.3389/fchem.2018.00261)
Supplement: Supplementary file 1 [file Presentation_1.PDF]

# A Plasma Biochemical Analysis of Acute Lead Poisoning in a Rat Model by Chemometrics-Based Fourier Transform Infrared Spectroscopy: An Exploratory Study

**Wenli Tian, Dan Wang, Haoran Fan, Lujuan Yang, and Gang Ma***

Key Laboratory of Medicinal Chemistry and Molecular Diagnosis of Ministry of Education, Key Laboratory of Analytical Science and Technology of Hebei Province, College of Chemistry and Environmental Science, Hebei University, Baoding 071002, China

*** Correspondence:** gangma@hbu.edu.cn

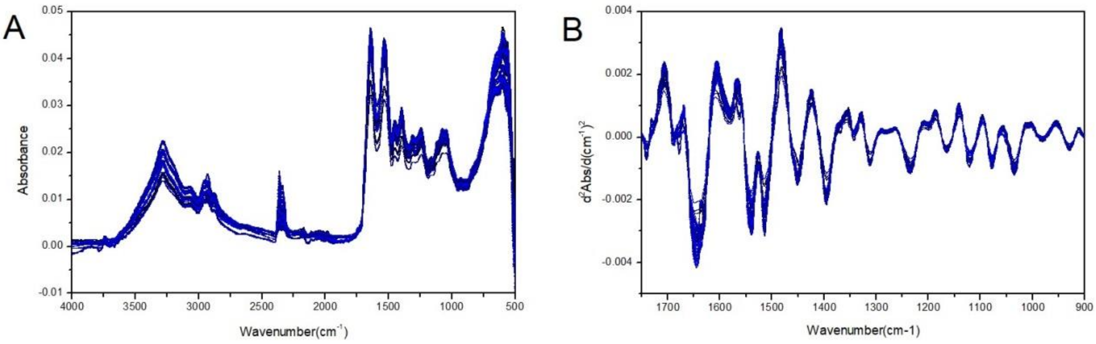


**FIGURE S1** Plasma FTIR spectra (A) and corresponding second derivatives (B) of the rat group with acute cadmium poisoning. The spectra were collected 24 hours after cadmium injection and there are a total of 82 spectra included in the figure.

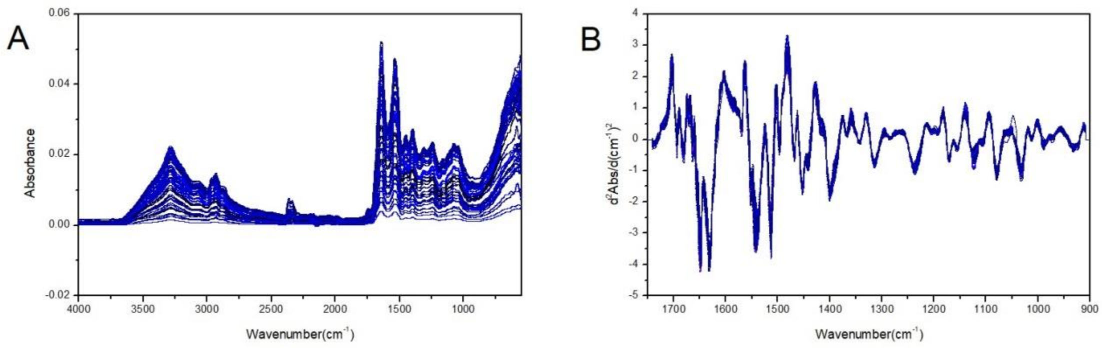


**FIGURE S2** Plasma FTIR spectra (A) and corresponding second derivatives (B) of the rat group with ALP. The spectra with ALP were collected 36 hours after lead injection and there are a total of 65 spectra included in the figure.

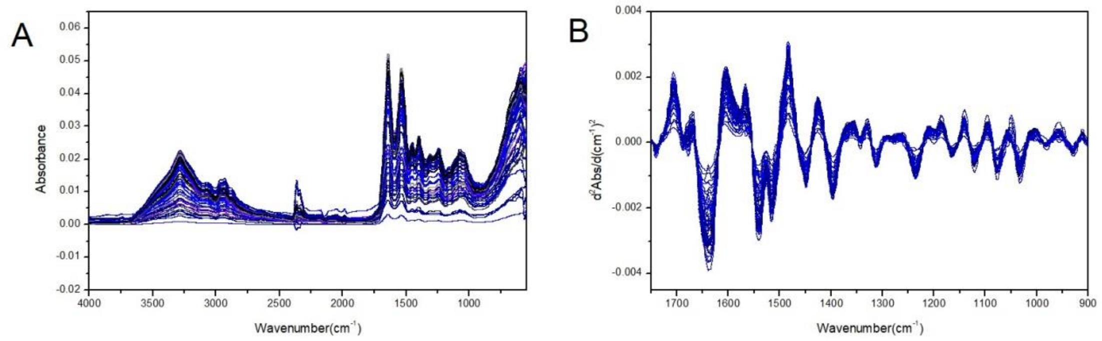


**FIGURE S3** Plasma FTIR spectra (A) and corresponding second derivatives (B) of the rat group with ALP. The spectra with ALP were collected 48 hours after lead injection and there are a total of 67 spectra included in the figure.
